# Protective role of Lagenaria Siceraria seed oil against furan-induced toxicity: Histopathological, biochemical, and molecular insights in male Albino rats

**DOI:** 10.1371/journal.pone.0322363

**Published:** 2025-05-14

**Authors:** Mona Zahran, Fatima S. Alaryani, Khlood M. El Bohi, Ehsan H. Abu Zeid, Mohamed H. Khairy, Aishah E. Albalawi, Reem H. Alhasani, Shatha G. Felemban, Reda Korany

**Affiliations:** 1 Department of Pharmacology, Faculty of Veterinary Medicine, Zagazig University, Zagazig, Egypt; 2 Department of Biological Sciences, University of Jeddah, College of Science, Jeddah, Saudi Arabia; 3 Department of Forensic Medicine and Toxicology, Faculty of Veterinary Medicine, Zagazig University, Zagazig, Egypt; 4 Department of Biology, Faculty of Science, University of Tabuk, Tabuk, Saudi Arabia; 5 Department of Biology, Faculty of Science, Umm Al-Qura University, Makkah, Saudi Arabia; 6 Medical Laboratory Sciences Department, Fakeeh College for Medical Sciences, Jeddah, Saudi Arabia; 7 Pathology Department, Faculty of Veterinary Medicine, Cairo University, Cairo, Egypt; 8 Faculty of Veterinary Medicine, Egyptian Chinese University, Cairo, Egypt; Alexandria University, EGYPT

## Abstract

This study aimed to assess the protective effects of *Lagenaria siceraria* seed oil (LSO) on fifty male Albino rats subjected to furan exposure. Furan (FU) is a small, heterocyclic compound present in the volatile fraction of various thermally processed foods and beverages. Rats were categorized into five groups, each comprising ten rats. Group 1 served as the control group, receiving corn oil. Group 2 received LSO (3 g/kg body weight orally) for 28 days. Rats in Group 3 (FU-exposed group) received an oral administration of FU at a dosage of 16 mg/kg body weight each day for 28 days. Rats in Group 4 (Therapeutic co-treated group) were administered both LSO and subsequent FU exposure according to the previously outlined dosage regimen for 28 days. Rats in Group 5 (Protective co-treated group) received LSO seed oil for 14 days as protection then received Fu at the same mentioned doses of Fu until the end of experiment. Rats administered FU and/or LSO gained noticeably more weight than the control group. LSO significantly decreased AST and LDH levels in both the protection and treatment groups as compared to the FU-only group. It also assisted in restoring testosterone and luteinizing hormone (LH) levels that were decreased by FU, especially in the protected group. LSO also reduced kidney damage markers and normalized biomarker levels when administered with FU. The LSO-only group demonstrated normal immune response markers, similar to the control group. By changing MDA levels and increasing SOD, GSH, and TAC levels, co-treatment with LSO enhanced liver health. The control and LSO groups displayed normal spleen structure, whereas the LSO/FU group had normal seminiferous tubules with mild edema and congestion. Overall, LSO demonstrated protective and therapeutic benefits against FU-induced damage in rats.

## Introduction

Various procedures in food processing, whether it’s thermal or non-thermal, lead to the formation of unwanted pollutants. The conversion of these contaminants into more dangerous metabolites can occur when enzymes are activated [1]. Thermal degradation of carbohydrates, for instance, results in the formation of certain furan (FU) compounds via non-enzymatic (Maillard) processes. Natural FU in food and drink is the result of a heat breakdown process that primarily involves carbohydrates, ascorbic acid, and polyunsaturated fatty acid components [2]. Furan (FU), a chemical pollutant, is generated during thermal [3] and non-thermal food processing, such as UV-C disinfection [4], and is found in many ready-to-eat meals. It’s used industrially in products like insecticides, solvents, and pharmaceuticals, and is also released from burning plastics, cigarette smoke, and engine exhaust [5]. Tobacco is a primary exposure source, with one cigarette containing up to 65 mg of FU [6]. Surveys by international food safety agencies revealed that FU levels exceed 100 ppb in vegetables like sweet potatoes, beans, and squash, with measurable amounts in most baby foods, baked goods like rusks and toasted bread, and canned meats [7].

The elevated levels of FU in roasted coffee beans are likely caused by the roasting process, which employs higher temperatures compared to other food processing methods [8]. As a result, people mostly absorb FU through coffee in their diet. In accordance with EFSA [7], the quantities of FU in food can range from a few micrograms per kilogram to seven thousand grams per kilogram. There have been studies that have followed the toxic effects of FU administration on many physiological systems, such as the immune system [9], reproductive system [10], liver and renal system [11], endocrine glands [12], and brain [13]. Cytochrome P450-mediated oxidation of the FU ring is the primary mechanism responsible for furan’s toxic effects. Among other biological components, this activity creates the toxic metabolite cis-butene-1,4-dial, which can bind irreversibly to DNA and protein [14].

BDA (cis-2-butene-1,4-dial), the main metabolite of FU, alters FU’s effects through various toxic pathways. It rapidly reacts with amino acids, nucleotides, biogenic amines, and glutathione, forming metabolites that signal FU exposure and potential harm [15]. Users of e-cigarettes, cannabis, or traditional tobacco show these metabolites in their urine, resulting from BDA reacting with protein nucleophiles. Their elevated FU mercapturic acid metabolite levels, compared to nonsmokers, suggest a higher risk of furan-related toxicity [16]. The International Agency for Research on Cancer labels furan (FU) as “possibly carcinogenic to humans” (Group 2B), raising global concern [17]. Studies showed that two years of FU exposure caused liver tumors (hepatocellular adenomas and carcinomas) in animals and increased cholangiocarcinomas in rats. While chronic toxicity and cancer risks from long-term, low-dose FU exposure have been studied in animals, the potential dangers and molecular impacts of rising FU exposure from diet and industrial sources remain uncertain and debated [18].

Recent research on nutraceuticals has focused on finding safe, natural alternatives to pharmaceuticals. Plant-derived nutraceuticals contain phyto complexes, while those from animals have various secondary metabolites [19,20]. Phenolic compounds, a major group of plant secondary metabolites, are found in about 8,000 plant-based nutraceuticals, with polyphenols showing great potential in medicine and food preservation despite their limitations [21]. Studies suggest that plant antioxidants, like extra virgin olive oil and Petroselinum crispum oil, may reduce kidney, liver, and nerve damage caused by xenobiotics in animals [22].

*Lagenaria siceraria*, more often known as bottle gourd, is a medicinal plant with several sections that have shown promise in the field of pharmaceuticals. It belongs to the Cucurbitaceae family and is characterized as a herbaceous climber. Because of its medicinal qualities, *Lagenaria siceraria* seed oil (LSO) is significant oil on the worldwide market. It is utilized in a wide variety of cosmetics, skincare products, and medicinal products for benign prostatic hyperplasia. In addition, a number of medications used to treat hirsutism, alopecia, excessive seborrhea, and acne can provide topical relief for migraine headaches [23]. According to Nigam et al. [24], LS seed oil has strong antioxidant and antibacterial properties against *Staphylococcus* and *E. coli*. Translucent, pale-yellow oil was extracted from bottle gourd seeds [25]. The fatty acid content of bottle gourd seeds is around 39.22%, palmitic acid (21.36%), oleic acid (63.32%), and linoleic acid (63.32%) are the three fatty acids that are most frequently recorded. Approximately 74.20% of its protein has biological value [26]. The current research looked at the protective functions of LSO against FU-induced various toxic actions in rats. This study is a groundbreaking exploration of how LSO protects male albino rats from FU-induced toxicity, examining histopathological, biochemical, and molecular effects. Unlike prior research on FU’s organ damage and other natural remedies, it’s one of the first to test LSO—a plant-based nutraceutical packed with bioactive compounds—as a defense against FU’s multi-organ harm. By combining these analyses, the study offers fresh, detailed insights into LSO’s potential as a natural counter to chemical pollutants like FU.

## Materials and methods

### Ethics statement

The ethics of animal use in research committee at Zagazig University in Egypt confirmed that all animals were cared for and handled in accordance with the guidelines set out by the National Institutes of Health in the United States.

### Tested chemical

*Lagenaria siceraria* (LSS) pale yellow and clear oil was obtained from Zagazig University, of Agriculture. Furan (C_4_H_4_O, ≥ 99% purity, molecular weight 68.07, CAS number 110–00–9) was acquired from Sigma-Aldrich Co., St. Louis, USA. Furan was diluted with maize oil and subsequently stored in brown glass vials at 4 °C for exposures was orally administered at a dosage of 16 mg/kg b.w/day as recommended by Alam et al. [9]. New solutions were created weekly as required. *Lagenaria siceraria* seed oil (LSO) was administered orally at a dosage of 3 g/kg body weight as recommended by Almohmadi et al. [59] and Shendge and Belemkar [27] [28].

### Gas chromatography–mass spectrometry analysis (GC-MS) of LSO bioactive chemical constituents

The gas chromatograph (7890B) and mass spectrometer detector (5977A) were part of the GC-MS system (Agilent Technologies) at the Central Laboratories Network, National Research Centre, Cairo, Egypt. The HP-5MS column, which had a 30 m x 0.25 mm internal diameter and a film thickness of 0.25 μm, was attached to the GC. The following temperature protocol was used in the analysis, which involved using hydrogen as the carrier gas at a flow rate of 2.0 mL/min with an injection volume of 2 µl. 5 minutes at 50 °C; 5 minutes at 100 °C with a hold; 10 minutes at 320°C with a rise of 10°C per minute.

A temperature of 280 °C and 320 °C were maintained for the injector. With a solvent delay of 6 minutes and a spectral range of 25–700 m/z, mass spectra were acquired by electron ionization (EI) at 70 eV. At 230°C, the mass temperature was higher than Quad’s 150°C. The spectrum fragmentation pattern was compared to data contained in the Wiley and NIST Mass Spectral Libraries in order to identify various components.

### Experimental animals

The Laboratory Animal Farm of the Faculty of Veterinary Medicine, Zagazig University, Egypt, supplied fifty male albino rats, each weighing 200 ± 15 g. The animals were housed in clean stainless steel cages under controlled conditions for two weeks before to usage, when they adjusted to a 12-hour light/dark cycle, relative humidity of 44–50%, and a temperature of 25 ± 2°C. Throughout the experiment, they were given fresh water and a typical diet and approved by Zu-IACUC comitte (Approval Number: Zu-IACUC/2/F/85/2022).

### Experimental design

Each of the five groups consisted of ten rats. Both before and after the experiment, the weight of the experimental animals will be recorded. Every day, an eye was kept on for any symptoms in the experimental animals that could be a result of the FU dosage.

- Group I (C): The control group that received corn oil.
- Group II (LSO): The rats received *Lagenaria siceraria* seed oil (3 g/kg b.wt. orall
- Group III: Oral administration of 16 mg/kg b.wt/day of furan was done to the rats in the furan-exposed group.
- The rats in Group IV, also known as the Therapeutic Co-treated group, were given LSO and FU at the same doses as in the previous section.
- Group V: (Protective co-treated group) rats were received LSO and concurrently with FU exposure at the previously described doses regimen

### Blood collection

Upon conclusion of the experiment, blood samples were obtained from the retro-orbital plexus of the eye of all subjects in each group. Clotted blood was utilized to obtain sera for biochemical analyses.

### Male fertility indices

Cauda epididymal sperm characteristics. The seminal picture was obtained by mincing the excised cauda epididymis finely with sterile scissors for about 1 minute, followed by incubation at 36°C for 5 minutes to ensure that all spermatozoa migrated from the epididymal tissue to the suspension. We used the methods described by Bearden and Fuquay [29] to estimate the concentration of sperm cells, and by Evans and Maxwell [30] to note any morphological abnormalities in the sperm.

### Steroid hormone analysis

Using commercial mice ELISA kits (MBS282195, MBS2502190, and MBS764675, respectively) and following the manufacturer’s instructions (My BioSource), the concentrations of serum testosterone, luteinizing hormone (LH), and follicle stimulating hormone (FSH) were measured.

### Biochemical analysis

#### Liver functions assay in serum.

Liver function tests were conducted on serum with rat ELISA kits acquired from My BioSource, San Diego, CA, USA. Alanine aminotransferase (ALT) (Catalog Number: MBS269614). Aspartate aminotransferase (AST), (Catalog No. MBS264975). Lactate dehydrogenase (LDH) levels were quantitatively assessed in serum utilizing colorimetric kits from Spinreact Co. (Santa Coloma, Spain). The assay was conducted in accordance with the kit’s instructions.

#### Kidney functions assay and protein profile in serum.

Following the manufacturer’s directions, colorimetric BIOMED Diagnostic-Egy Chem kits (Badr city, Egypt) were utilized to quantitatively assess the protein profile (total protein and albumin) and renal damage markers (creatinine and urea). The globulin level and albumin to globulin ratio were measured.

#### Lipid profile.

Commercial kits were provided by the Bio diagnostic Company (Giza, Egypt) to measure triglycerides, total cholesterol, high-density lipoprotein, cholesterol, and low-density lipoprotein in serum.

#### Immune response markers.

The rat ELISA kits utilized in this study were obtained from My BioSource in San Diego, USA. This item is listed under catalog number MBS2510638. The manufacturer-recommended protocols were followed for the quantitative assessment of these biomarkers. Ellis’s approach [31] was used to spectrophotometrically assess the serum’s lysozyme activity by lysing freeze-dried particles of Micrococcus lysodeikticus. The nitrogen oxide (NO) concentrations in the blood were measured using a calorimetric method.

#### Evaluation of antioxidant status in liver, testes and spleen tissue homogenates.

Assay kits from Bio Diagnostic Co., Egypt, were utilized to quantify: Reduced glutathione (GSH) (Cat. No. GR2511), Superoxide dismutase (SOD) (Cat. No. SD2521), Catalase (CAT) (Catalog No. CA2517), Total antioxidant capacity (TAC) (Catalog No. TA 2513), as specified by the makers.

#### Evaluation of oxidative stress in liver, spleen and testes tissue homogenates.

Malondialdehyde (MDA), a lipid peroxidation product, was measured using Bio Diagnostic (Dokki-Giza, Egypt) (Catalog No. MD2529).

#### Evaluation of inflammatory biomarkers.

Rat ELISA kits were utilized and provided by My BioSource, San Diego, USA. The purchased kits were Tumor necrosis factor-alpha (TNF-α) (Catalog No. MBS267737), IL6 (Catalog Number MBS2508830), IL10 (Catalog Number MBS243214). The quantitative assessment of these biomarkers was conducted in accordance with the manufacturer’s prescribed protocols.

#### Real-time quantitative polymerase chain reaction analysis of genes encoding inflammation and apoptosis).

In order to generate first-strand cDNA, we used a QuantiTect Reverse Transcription kit from Qiagen in Heidelberg, Germany. The rats’ liver and kidneys were used as sources of total RNA, which was isolated using the RNeasy Mini Kit from Qiagen. The QuantiTect SYBR Green PCR kits from Qiagen were used in a Rotor-Gene Q cycler from Qiagen, together with forward and reverse primers for each gene. The process was carried out in Heidelberg, Germany. Following real-time PCR, melting curves were plotted to show how each target gene product was amplified. The primers’ amplification efficiency was determined using a standard curve test. Three separate samples were evaluated for every category.

The relative fold levels in the mRNA expression of the target genes that were studied were determined using the comparative 2^−ΔΔCt^ method, with glyceraldehyde-3-phosphate dehydrogenase serving as an internal reference to standardize the levels of target gene expression. This method was described by Livak and Schmittgen [32].

#### Tissues samples collection and preparation.

Histological preparations involve preserving samples of the liver, kidney, testes, and spleen in neutral buffered formalin (NBF) during the autopsy. By anesthetizing the mice and secure the rat dorsally on a dissection board, limbs pinned. Made a midline abdominal incision from pelvis to sternum. Organs/tissues placed in biohazard bags, autoclave if infectious, and dispose via regulated medical waste (incineration). In order to analyze the gene expression of certain genes in tissues, a collection of tissue samples will be kept in liquid nitrogen at a temperature of -80 °C. After being washed with ice-cold phosphate buffer saline, another set of tissue samples was homogenized in 9 liters of the same solution. Spin the mixture of tissues at 10,000 rpm (4°C) for 10 minutes. Biochemical analysis required the collection of the supernatant. For seminal analysis, one testis’s cauda epididymis was removed and placed in a sterilized petri dish with normal saline that had been warmed to 37°C.

#### Histological methods.

Prior to examination under a light microscope (Olympus BX51 Microscope), the fixed tissue specimens underwent histological preparations, which included staining with hematoxylin and eosin (H&E) [33].

#### Immunohistochemical analysis.

To deactivate the peroxidases, tissue sections were first treated with 3% hydrogen peroxidase for 10 minutes after being deparaffinized with xylene. After soaking the samples in a citrate solution with 10 mM concentration for 30 minutes at 121°C, the antigen was extracted. The slices were incubated overnight at 4 °C in phosphate buffered saline after being blocked for 20 minutes in 5% normal serum. They were then incubated with a goat anti-rabbit IgG biotin-conjugated secondary antibody (1:2000; sc 2040; Santa Cruz Biotechnology, Inc.) for 20 minutes at 32 °C after three consecutive washing with phosphate-buffered saline.

The sections were counterstained with hematoxylin as a contrasting agent and evaluated after additional incubation with horseradish peroxidase-labeled streptavidin. Antibody binding was then detected using diaminobenzidine.

### Statistical analysis

In order to conduct the statistical analysis, SPSS (SPSS Inc., Chicago, IL, USA, version 11.5) was utilized. When the data was regularly distributed, we compared the groups’ parameters using one-way analysis of variance (ANOVA). Prior to performing any statistical analysis, the Shapiro-Wilk test was utilized to check for normality. Multiple comparisons were conducted using Tukey’s post hoc test. In addition, Levene’s test results demonstrated that the groups’ variances were equal. Mean plus or minus one standard deviation (SD) was the way the results were presented. Statistical significance was defined as a p-value lower than 0.05.

## Results

### GC-MS profiling of the *Lagenaria siceraria* (LS) oil

Table 1 displays the chemical composition of the LSO, with the compounds listed according to the sequence in which they were eluted on the HP-5MS column. By analyzing the mass spectra and retention indices of 37 potential components of this oil, the GC-MS method was able to define and identify it.

**Table 1:** Bioactive chemical constituents assigned in LSO by GC-MS analysis.

No.	Bioactive chemical constituents	C	Retention Time (min)	Area %
1.	Butyric acid	C4:0	5.099	0.41
2.	caproic acid	C6:0	5.784	0.52
3.	Caprylic acid	C8:0	8.055	2.89
4.	Capric acid	C10:0	12.101	3.7
5.	Undecanoic acid	C11:0	14.707	1.92
6.	Lauric acid	C12:0	17.562	3.96
7.	Tridecanoic acid	C13:0	20.522	2.01
8.	Myristic acid	C14:0	23.531	4.13
9.	Myristoleic acid	C14:1	25.199	1.99
10.	Pentadecanoic acid	C15:0	26.494	2.07
11.	cis-10-pentadecenoic acid	C15:1	28.116	2.38
12.	Palmitic acid	C16:0	29.43	6.46
13.	Palmitoleic acid	C16:1	30.59	2.1
14.	Margaric acid	C17:0	32.231	2.16
15.	cis-10-Heptadecenoic acid	C17:1	33.349	2.14
16.	Stearic acid	C18:0	35.002	4.34
17.	Elaidic acid	C18:1	35.539	2.17
18.	Oleic acid	C18:1	35.836	4.44
19.	Linolelaidic acid	C18:2	36.813	2.19
20.	Linoleic acid	C18:2	37.534	2.64
21.	gamma-Linolenic acid	C18:3	38.732	2.21
22.	Linolenic acid	C18:3	39.639	2.21
23.	Arachidic acid	C20:0	40.238	4.45
24.	cis-11-Eicosenoic acid	C20:1	40.922	2.26
25.	cis-11,14-Eicosadienoic acid	C20:2	42.49	2.21
26.	Heneicosanoic acid	C21:0	42.708	2.23
27.	Homo-γ-linolenic acid	C20:3	43.541	2.25
28.	Arachidonic acid	C20:4	44.331	2.06
29.	cis-11,14,17-Eicosatrienoic acid	C20:3	44.421	2.17
30.	Behenic acid	C22:0	45.161	4.6
31.	Erucic acid	C22:1	45.75	2.8
32.	EPA	C20:5	46.387	2.2
33.	cis-13,16-Docosadienoic acid	C22:2	47.157	2.27
34.	Tricosanoic acid	C23:0	47.469	2.3
35.	Lignoceric acid	C24:0	49.803	4.61
36.	Nervonic acid	C24:1	50.3	2.34
37.	DHA	C22:6	51.624	2.21

Classification of the chemical components allowed for their identification; the majority of these components are involved in the oil’s essential biological actions. The most prominent components were found to be palmitic acid (6.46%), behenic acid (4.6%), lignoceric acid (4.61%), arachidic acid (4.45%), oleic acid (4.44%), stearic acid (4.34%), and myristic acid (4.13%). Additionally, nervonic acid (2.34%), caprylic acid (2.89%), lauric acid (3.96%), tridecanoic acid (2.01%), linolenic acid (2.21%), and others were detected. Throughout the study duration, the rats in the control group, as well as those administered LSO and those receiving protective/therapeutic co-treatment, were continuously normal. Conversely, dullness, drowsiness, and coarse hair coats have been noted in another trial group, particularly in the FU-treated cohort. No fatalities have been documented in the treated groups. Rats received furan and or LSO showed a significant increase in body weight gain compared to the control group (Table 3).

### Effect of LSO supplementation on male fertility of rat exposed to FU

#### Cauda epididymal sperm characteristics (epididymal sperm concentration, viability, and abnormalities).

Post-exposure, a notable decline in sperm content and viability was recorded in the FU-exposed rats. The protective group exhibited a considerable enhancement in sperm count and viability compared to the Fu-exposed group, however no changes were observed in the treatment group.

The incidence of epididymal sperm abnormalities in the FU-treated groups was substantially greater than that observed in the control group. Regrettably, the introduction of LSO in both co-administered groups did not significantly alter the reported levels of anomalies compared to the FU-exposed value (Table 2).

**Table 2:** Male fertility indices in rats in response to LSO administration and/or FU treatment.

Parameters	Control	LSO	FU	FU/LSO	LSO/FU
Epididymal seminal picture					
Sperm count (10^6^)	76.33 ± 3.38^a^	81.66 ± 1.76^a^	45.66 ± 1.45^c^	45.00 ± 1.52^c^	56.33 ± 1.20^b^
Sperm motility (%)	63.66 ± 1.76^a^	67.33 ± 2.90^a^	47.66 ± 2.40^c^	53.33 ± 3.17^bc^	58.66 ± 0.88^ab^
Sperm abnormalities (%)	7.33 ± 0.33^b^	7.00 ± 0.57^b^	11.33 ± 0.33^a^	9.66 ± 0.33^a^	9.33 ± 0.66^a^
Hormonal analysis					
Testosterone (ng/mL)	1.46 ± 0.04^ab^	1.56 ± 0.06^a^	1.23 ± 0.03^b^	1.29 ± 0.04^b^	1.45 ± 0.06^ab^
FSH (ng/ mL)	0.23 ± 0.02 ^a^	0.26 ± 0.02 ^a^	0.18 ± 0.03^b^	0.17 ± 0.02^b^	0.17 ± 0.01^b^
LH (ng/ mL)	0.18 ± 0.03^ab^	0.24 ± 0.02^a^	0.12 ± 0.01^b^	0.15 ± 0.03^ab^	0.17 ± 0.02^ab^

Means within the same row with differing superscripts (a, b, c, d) are deemed statistically different (*P* < 0.05).

Steroid hormone analysis (Testosterone, FSH and LH levels). Rats exposed to FU exhibited a notable reduction in serum testosterone and LH levels compared to the control group. Conversely, the LSO dose reinstated the FU-induced decline in testosterone levels relative to the FU-exposed group, especially in the rats of the protected group. Additionally, LSO was shown to reinstate LH levels in both protective and therapeutic protocols, in contrast to the FU-exposed cohort. Following FU exposure and/or LSO inclusion, FSH hormone levels exhibited no statistically significant difference across all groups (*P* < 0.05) (Table 2).

### Effect of LSO supplementation on tissue injury markers of rat exposed to FU

#### Liver injury markers (serum ALT, AST) and lactate dehydrogenase (LDH).

In rats exposed to furan, the activity of hepatic enzymes ALT, AST, and LDH were significantly elevated (*P* < 0.05) compared to the control rats. Significant restoration of the previous biomarkers was accomplished with LSO supplementation, especially in the group 5 protective group. ALT levels did not exhibit a significant decrease in the treatment group, whereas a significant fall was observed in the protective group. Conversely, AST markedly decreased significant in both the protective and treatment groups. Furthermore, the LDH levels were significantly reduced in both co-exposed groups relative to the FU-exposed group, indicating a trend towards normalization. Nonetheless, it did not correspond with that of the control (Table3).

#### Renal injury markers.

FU exposure demonstrated a substantial elevation in the concentrations of urea, uric acid, and creatinine relative to the control group. The concurrent administration of LSO with FU markedly reduced these indicators in both the preventive and therapeutic groups. The biomarker levels in both co-treated groups were adjusted and standardized to the control level (Table 3).

#### Serum protein profile.

The sera of rats exhibited no variations in protein concentration and its fractions (globulin, albumin) across all experimental groups (Table 3).

#### Serum lipid profile.

FU exposure markedly increased the levels of total cholesterol and LDL in comparison to the control group. The increase was regulated by the administration of LSO, normalizing in both protective and therapeutic regimens, and markedly differing from the Fu-exposed group. The levels of TG, HDL, and VLDL exhibited no significant variations across all treated groups (Table 3).

### Effect of LSO supplementation on immune response of rat exposed to FU

A notable reduction was observed in IgM, NO levels, and lysozyme activity in the serum of FU-exposed rats, in comparison to the control rats’ values. Similar to the control group, the LSO-only treated group has exhibited normal levels of the aforementioned markers. A notable alteration in IgM levels was observed just in the protective group, in contrast to the therapeutic co-administered group as compared to FU-exposed rats. LSO supplementation significantly altered NO levels in both co-treated groups, with a sharper effect observed in the therapeutic regimen, which showed a non-significant difference compared to the control group. Furthermore, the co-treatment of FU-exposed rats with LSO did not produce a substantial alteration in lysozyme activity, exhibiting a non-significant difference when compared to the FU-intoxicated group in both regimens (Table 4).

**Table 3:** Body weight changes, tissue injury biomarkers, lipid and protein profiles in rats in response to LSO administration and/or FU treatment.

Parameters	Control	LSO	FU	FU/LSO	LSO/FU
Body weight changes (gm)	16.66 ± 1.20^c^	50.00 ± 4.58^ab^	39.33 ± 1.76^b^	59.33 ± 5.60^a^	49.33 ± 4.84^ab^
ALT (U/L)	21.00 ± 1.15^c^	22.00 ± 1.15^c^	35.00 ± 1.73^a^	31.00 ± 0.57^ab^	29.33 ± 0.33^b^
AST (U/L)	316.00 ± 4.04^bc^	301.33 ± 10.71^c^	372.33 ± 6.93^a^	340.67 ± 4.84^b^	330.67 ± 2.02^b^
LDH (U/L)	180.67 ± 9.61^c^	171.10 ± 3.60^c^	303.33 ± 13.73^a^	261.00 ± 8.02^b^	230.67 ± 5.55^b^
Urea (mg/dl)	41.53 ± 1.87^b^	41.46 ± 1.95^b^	52.46 ± 2.58^a^	46.96 ± 1.75^a^	41.33 ± 1.11^b^
Creatinine (mg/dl)	0.27 ± 0.03^b^	0.26 ± 0.02^b^	0.40 ± 0.03^a^	0.27 ± 0.01^b^	0.28 ± 0.01^b^
Uric acid (mg/dl)	1.99 ± 0.05^ab^	1.83 ± 0.13^b^	2.23 ± 0.02^a^	2.08 ± 0.03^ab^	1.96 ± 0.03^ab^
Total protein (gm/dl)	6.60 ± 0.20	6.76 ± 0.16	6.38 ± 0.16	6.26 ± 0.13	6.48 ± 0.18
Albumin (gm/dl)	4.46 ± 0.12	4.55 ± 0.08	4.20 ± 0.10	4.28 ± 0.06	4.37 ± 0.05
Globulin (gm/dl)	2.13 ± 0.10	2.19 ± 0.09	2.18 ± 0.05	1.98 ± 0.08	2.10 ± 0.13
Cholesterol (mg/dl)	84.33 ± 5.36^b^	67.33 ± 11.85^b^	105.67 ± 3.52^a^	75.66 ± 4.80^b^	81.00 ± 3.51^b^
Triglycerides (mg/dl)	63.66 ± 1.76	62.00 ± 3.21	68.33 ± 5.48	51.00 ± 1.15	63.33 ± 6.56
LDL (mg/dl)	28.33 ± 2.77^b^	24.93 ± 4.47^b^	48.33 ± 5.96^a^	28.80 ± 1.31^b^	25.33 ± 3.29^b^
HDL (mg/dl)	44.66 ± 1.20	34.00 ± 5.00	39.33 ± 0.33	35.33 ± 2.33	36.33 ± 2.02
VLDL (mg/dl)	13.00 ± 0.61	12.40 ± 0.64	13.00 ± 1.30	10.20 ± 0.23	12.66 ± 1.31

Means within the same row with differing superscripts (a, b, c, d) are deemed statistically different (*P* < 0.05).

LSO: *Lagenaria siceraria seed oil*, FU: Furan-exposed group, LSO/FU: Prophylaxis co-treated group, FU/LSO: Therapeutic co-treated group.

**Table 4:** Immune responses in rats in response to LSO administration and/or FU treatment.

Parameters	Control	LSO	FU	FU/LSO	LSO/FU
IgM (mg/dl)	37.33 ± 1.76^a^	38.33 ± 1.45^a^	24.33 ± 1.20^b^	26.00 ± 2.08^b^	33.33 ± 2.40^a^
NO (µmol/L)	17.16 ± 0.52^ab^	17.86 ± 0.84^a^	11.11 ± 0.87^c^	15.44 ± 0.27^ab^	14.32 ± 0.24^b^
Lysozyme activity (U/ mL)	578.33 ± 1.45^a^	597.66 ± 9.76^a^	448.33 ± 16.50^b^	470.66 ± 22.73^b^	496.33 ± 11.96^b^

Means within the same row with differing superscripts (a, b, c, d) are deemed statistically different (*P* < 0.05).

### Effect of LSO supplementation on oxidative stress/antioxidant status markers in different tissues of rat exposed to FU

#### Oxidative stress/antioxidant status markers in liver tissue.

An elevated MDA level was seen in the hepatic tissue of FU-exposed rats, in comparison to control rats. Co-treatment with LSO resulted in a notable alteration of hepatic MDA levels alone in the protective group, while no such effect was observed in the therapeutically co-administered group when compared to FU-exposed rats. Despite substantial modulation by LSO supplementation, the level remained considerably elevated in comparison to the control group.

The aforementioned elevation in MDA levels correlated with a reduction in the estimated antioxidant indices (SOD, CAT activity, GSH content, and TAC) in the livers of rats subjected to FU, in comparison to the control group, suggesting that FU induces oxidative injury. Furthermore, LSO supplementation was observed to partially enhance the diminished levels of SOD, GSH, and TAC when delivered concurrently with FU, with more pronounced effects in the protective co-treatment group compared to the therapeutic group. The modification of CAT activity in both co-administered groups resulted in a non-significant difference compared to FU-exposed hepatic tissue and did not reach the control value.

In the protective co-treated group, SOD activity was adjusted to be statistically indistinguishable from the control group, but, in the therapeutic co-treated group, the improvement remained considerably lower than that of the control group. The hepatic tissue of the protective approach exhibited a minor increase in GSH and TAC content, which did not reach normal levels, but no changes were observed in the treatment regimen (Table 5).

#### Oxidative stress/antioxidant status markers in spleen tissue.

The administration of FU increased the MDA levels in spleen tissue relative to the control group. The co-administration of LSO with FU exposure mitigated the elevation of the oxidative stress marker (MDA) compared to the BF-exposed group, restoring control values in both groups.

Conversely, the activities of SOD and CAT, as well as the levels of TAC and GSH in both co-treated groups, appeared to be comparatively elevated relative to their respective controls. Exposure of rats to FU resulted in a substantial reduction in all antioxidant parameters compared to the control group. Co-treatment with LSO and FU enhanced the inhibited enzyme activity and reduced glutathione and TAC levels, in contrast to the FU group. The control values for all variables were obtained in both the protective and therapeutic co-treated groups (Table 6).

#### Oxidative stress/antioxidant status markers in testicular tissue.

Exposure to FU resulted in a marked increase in the MDA levels within the testes. Both the protective (LSO/FU) and therapeutic (FU/LSO) groups exhibited a notable reduction in the increased MDA levels; nevertheless, these levels remained considerably distinct from the control group.

Furthermore, FU exposure markedly reduced the levels of testicular TAC, GSH, and the activity of SOD and CAT, in comparison to the control group. The reduction in TAC was markedly enhanced by the co-administration of LSO in a protective regimen, although it exhibited no significant change in the therapeutically co-treated group compared to the FU-exposed group. Conversely, the reduced activities of CAT and SOD, together with GSH levels, were adjusted in the testes to show no significant difference compared to control values, however remained low in both co-treated groups (Table 7).

### Effect of LSO supplementation on inflammatory markers in serum of rat exposed to FU

The concentrations of TNF-α and IL-6 were markedly increased in the FU-exposed rats relative to the control rats. The co-administration of LSO with FU markedly reduced the increase of these indices, especially in the protective co-treated rats, more so than in the therapeutic regimen, as compared to rats exposed just to FU. The recorded modulation normalized the inflammatory response to the control value, except for the IL6 level in the therapeutically co-treated group, which did not reach the control value. Conversely, FU exposure markedly reduced the IL10 levels in comparison to the control group. The reduction was enhanced by the administration of LSO in both protective and therapeutic regimens, in comparison to the FU-exposed group (Table 8).

**Table 5:** Oxidative stress/antioxidant status indices in liver of rats in response to LSO administration and/or FU treatment.

Parameters	Control	LSO	FU	FU/LSO	LSO/FU
MDA (nmol/gm)	97.23 ± 1.59^c^	80.26 ± 0.80^d^	121.53 ± 2.02^a^	113.80 ± 4.46^ab^	109.00 ± 3.50^b^
TAC mM/l	0.35 ± 0.03^ab^	0.37 ± 0.02^a^	0.22 ± 0.02^c^	0.24 ± 0.01^c^	0.28 ± 0.01^bc^
SOD (U/gm)	19.80 ± 0.65^ab^	21.18 ± 1.26^a^	14.01 ± 1.04^c^	17.30 ± 0.45^b^	18.53 ± 0.37^ab^
CAT(U/gm)	5.06 ± 0.06^a^	5.20 ± 0.25^a^	3.68 ± 0.12^b^	4.26 ± 0.42^ab^	4.69 ± 0.34^ab^
GSH (mg/gm)	160.23 ± 6.84^a^	172.23 ± 6.49^a^	130.33 ± 0.94^b^	138.13 ± 2.53^b^	151.10 ± 5.21^ab^

Means within the same row with differing superscripts (a, b, c, d) are deemed statistically different (*P* < 0.05).

**Table 6:** Oxidative stress/antioxidant status indices in spleen of rats in response to LSO administration and/or FU treatment.

Parameters	Control	LSO	FU	FU/LSO	LSO/FU
MDA (nmol/gm)	90.35 ± 3.54^bc^	82.63 ± 2.40^c^	110.93 ± 4.34^a^	96.80 ± 1.98^b^	96.00 ± 2.15^b^
TAC (mM/l)	0.33 ± 0.02^ab^	0.36 ± 0.03^a^	0.19 ± 0.02^c^	0.23 ± 0.02^bc^	0.26 ± 0.01^abc^
SOD (U/gm)	18.74 ± 0.79^b^	20.29 ± 0.40^a^	13.93 ± 1.50^b^	16.41 ± 0.59^ab^	16.14 ± 0.97^ab^
CAT(U/gm)	4.59 ± 0.30^a^	4.89 ± 0.23^a^	3.28 ± 0.34^b^	4.02 ± 0.02^ab^	4.06 ± 0.03^ab^
GSH (mg/gm)	124.90 ± 2.76^ab^	131.43 ± 1.33^a^	110.63 ± 4.71^b^	121.53 ± 0.93^ab^	120.00 ± 5.04^ab^

Means within the same row with differing superscripts (a, b, c, d) are deemed statistically different (*P* < 0.05).

**Table 7:** Oxidative stress/antioxidant status indices in testes of rats in response to LSO administration and/or FU treatment.

Parameters	Control	LSO	FU	FU/LSO	LSO/FU
MDA (nmol/gm)	116.86 ± 2.39^b^	114.96 ± 2.52^b^	136.20 ± 4.01^a^	124.63 ± 1.24^ab^	126.80 ± 2.82^ab^
TAC mM/l	0.17 ± 0.01^ab^	0.19 ± 0.01^a^	0.12 ± 0.01^c^	0.14 ± 0.01b^c^	0.15 ± 0.00^abc^
SOD (U/gm)	13.80 ± 0.55^b^	16.57 ± 0.88^a^	11.00 ± 0.45^c^	12.34 ± 0.26b^c^	12.37 ± 0.23^bc^
CAT(U/gm)	3.73 ± 0.15^ab^	4.12 ± 0.03^a^	2.99 ± 0.05^c^	3.34 ± 0.08^bc^	3.32 ± 0.17^bc^
GSH (mg/gm)	131.13 ± 3.98^ab^	137.50 ± 3.23^a^	119.83 ± 0.95^c^	122.66 ± 0.61^bc^	126.06 ± 0.12^bc^

Means within the same row with differing superscripts (a, b, c, d) are deemed statistically different (*P* < 0.05).

**Table 8:** Inflammatory markers in rats in response to LSO administration and/or FU treatment.

Parameters	Control	LSO	FU	FU/LSO	LSO/FU
TNF-α (pg/ mL)	58.33 ± 2.33^b^	54.33 ± 1.45^b^	70.66 ± 1.76^a^	58.66 ± 2.40^b^	56.66 ± 1.66^b^
IL6 (pg/ mL)	115.67 ± 3.48^c^	116.67 ± 1.76^c^	150.33 ± 3.17^a^	138.00 ± 1.15^b^	124.67 ± 3.52^c^
IL10 (pg/ mL)	64.66 ± 2.02^a^	59.66 ± 1.20^ab^	50.66 ± 3.17^b^	61.66 ± 3.17^ab^	55.66 ± 2.90^ab^

Means within the same row with differing superscripts (a, b, c, d) are deemed statistically different (*P* < 0.05).

### Histopathological finding

#### Testes.

**Fig 1:**
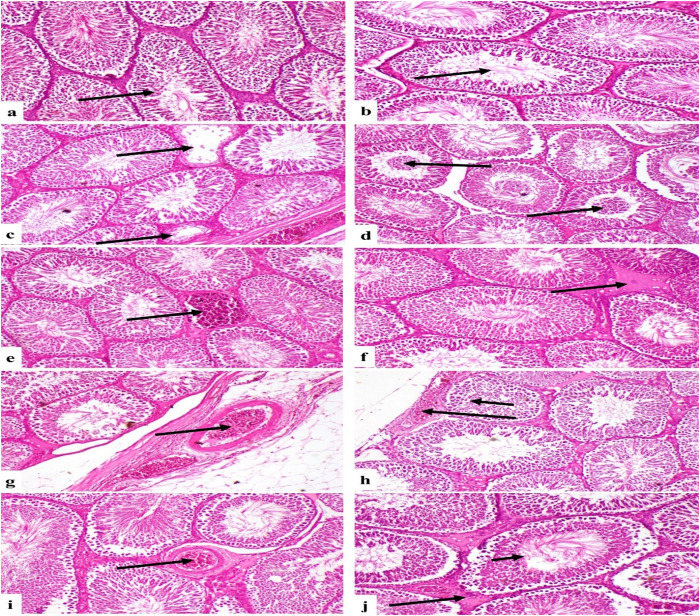
Photomicrograph of rat testes. (a) and (b) Control and LSO groups respectively showing normal histological structure of seminiferous tubules (arrow). (c) Fu-treated group showing degeneration of seminiferous tubules (arrows). (d) Fu-treated group showing degeneration and coagulation of sperms (arrows). (e) Fu-treated group showing congestion of interstitial blood vessel (arrow). (f) Fu-treated group showing edema of interstitial tissue (arrow). (g) Fu-treated group showing congestion and edema in tunica albuginea (arrow). (h) Group treated with Fu/ LSO showing necrosis of few seminiferous tubules (short arrow) and moderate congestion of tunica albuginea blood vessels (long arrow). (i) Group treated with Fu/ LSO showing moderate interstitial edema and congestion (arrow). (j) LSO/Fu group showing normal seminiferous tubules (short arrow) and mild interstitial edema and congestion (long arrow). (H&E X200).

The Control and LSO groups exhibited the typical histological architecture of seminiferous tubules and interstitial tissue (Fig 1a and b). The fu-treated group exhibited testicular degeneration characterized by a diminished quantity of spermatogenic cells within the seminiferous tubules, accompanied by degeneration and necrosis of other spermatogenic cells (Fig 1c). This group also demonstrated degeneration and coagulation of sperm within the seminiferous tubules (Fig 1d), congestion of interstitial blood vessels (Fig 1e), and edema of the interstitial tissue between seminiferous tubules (Fig 1f). Additionally, congestion and edema were observed in the tunica albuginea (Fig 1g). The cohort administered Fu/ LSO exhibited degeneration and necrosis in several seminiferous tubules, moderate congestion of the tunica albuginea blood vessels (Fig 1h), as well as moderate interstitial edema and congestion (Fig 1i). The LSO/Fu group exhibited normal seminiferous tubules accompanied by modest interstitial edema and congestion (Fig 1j).

#### Spleen.

**Fig 2:**
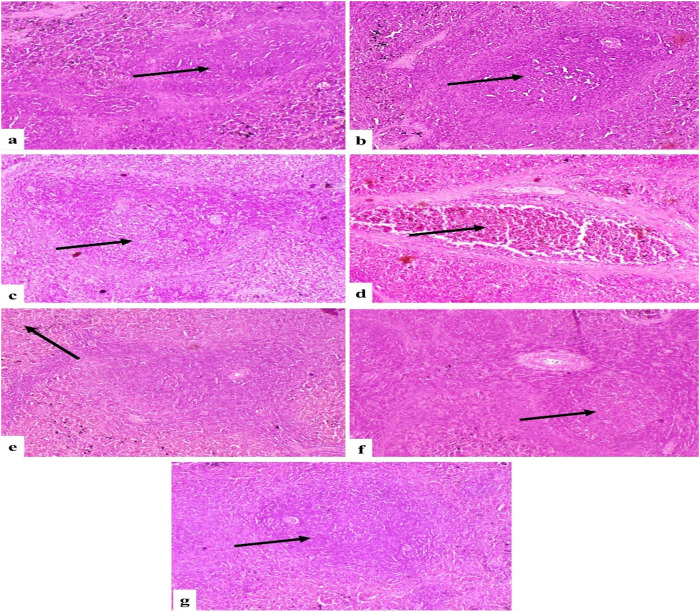
Photomicrograph of rat spleen. (a) and (b) Control and LSO groups respectively showing normal histological structure of red and white pulp (arrow). (c) Fu-treated group showing lymphocytic depletion of lymphoid follicles (arrow). (d) Fu-treated group showing congestion of splenic blood vessels (arrow). (e) Fu-treated group showing splenic hemorrhage (arrow). (f) Group treated with Fu/ LSO showing moderate lymphoid depletion (arrow). (g) LSO/Fu group showing normal lymphoid follicle (arrow). (H&E X200).

The Control and LSO groups exhibited the typical histological architecture of red and white pulp (Fig 2a and b). The fu-treated group exhibited necrosis and lymphocytic depletion of lymphoid follicles (Fig 2c), congestion of splenic blood vessels (Fig 2d), and splenic bleeding (Fig 2e). The cohort administered Fu/LSO exhibited moderate lymphoid depletion and bleeding (Fig 2f). The LSO/Fu group exhibited almost normal lymphoid follicles and minimal bleeding (Fig 2g).

#### Histopathological lesion score.

Recorded lesions in testes and spleen were scored according to their severity in Table 9.

**Table 9:** Lesion scoring of histopathological changes in testes and spleen of all treated groups.

Lesions	C	L.S.O.	Fu	Fu/ L.S.O.	L.S.O./Fu
Testes Testicular degeneration Congestion of interstitial blood vessels Edema of interstitial tissue Congestion of tunica albuginea blood vessels Spleen Lymphoid depletion Splenic vascular congestion Splenic hemorrhage	0 0 0 0 0 0 0	0 0 0 0 0 0 0	3 3 3 3 3 3 3	1 2 2 2 2 2 2	0 1 1 1 0 1 1

The scoring system was established as follows: score 0 indicates the absence of lesions in all rats in the group (n = 5), score 1 denotes lesions in less than 30%, score 2 represents lesions between 30% and 50%, and score 3 signifies lesions exceeding 50%.

#### Immunohistochemical findings for Caspase-3 and TNF-α expression.

**Fig 3:**
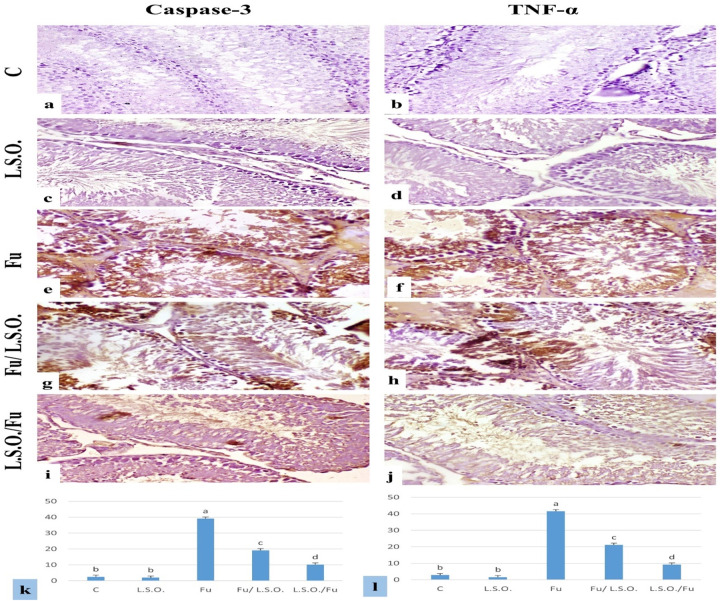
Immunostaining of caspase-3 and TNF- **α** **in rat testes,** (a)**-(d) control and LSO groups respectively showing very weak or no immuno-expression.** (e), and (f) Fu-treated group showing strong positive immune expression. (g), and (h) Fu/ LSO group showing moderate positive expression of caspase-3 and TNF-α. (i), and (j) LSO/Fu showing weak caspase-3 and TNF-α immune-reactive cells (Caspase-3 and TNF-α X200). (k), and (l) area % of caspase-3 and TNF-α expression in testes (data were expressed as mean ±SE, different letters (a, b, c & d) indicating significant differences at p < 0.05).

**Fig 4:**
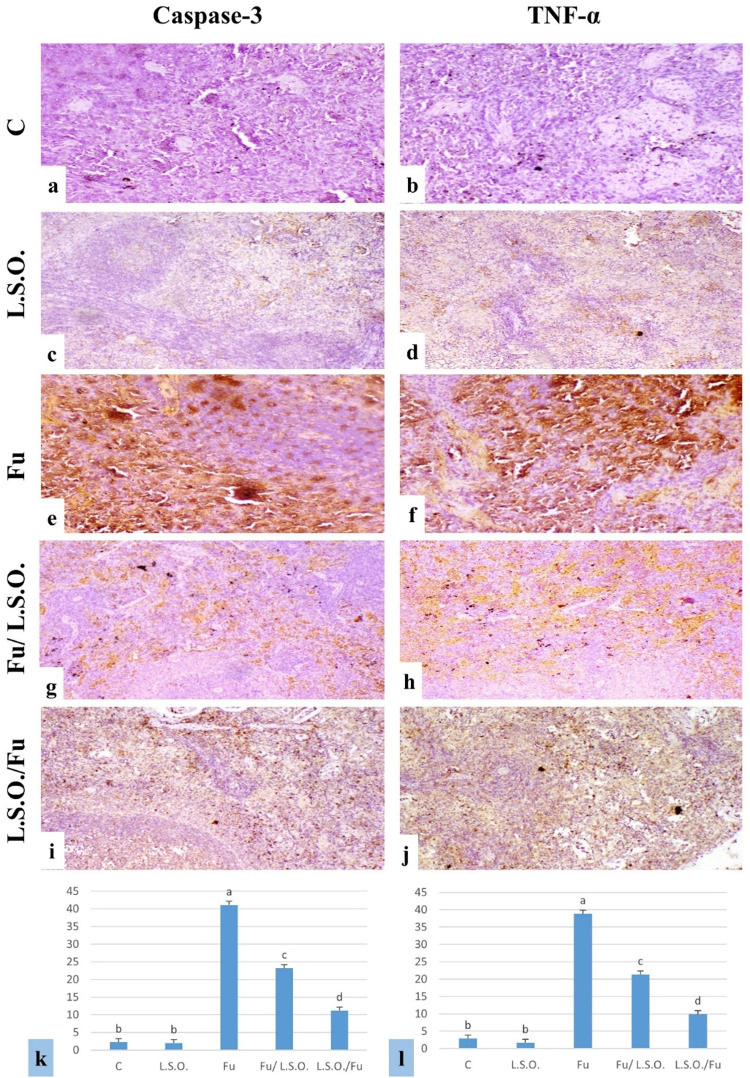
Immunostaining of caspase-3 and TNF- **α** **in rat spleen,** (a)**-(d) control and LSO groups respectively showing very weak or no caspase-3 and TNF-α** **immune-reactive cells in the spleen.** (e) and (f) Fu-treated group showing strong positive immune expression. (g), and (h) Fu/ LSO group showing moderate positive expression. (i), and (j) LSO/Fu showing weak caspase-3 and TNF-α immune-reactive cells (Caspase-3 and TNF-α X200). (k), and (l) I**mmunostaining expression area % of caspase-3 and TNF-α in the spleen (data were expressed as mean ±SE, different letters (a, b, c & d) indicating significant differences at** ****P****** < 0.05).**

The expression levels of caspase-3 and TNF-α percentage area in the testes and spleen are depicted in Figs 3 and 4(k and l). The expression of caspase-3 and TNF-α in the testes and spleen exhibited little or absent immune-reactive cells in both the control and L.S.O. groups (Figs 3 and 4a–d). The testes and spleen of the Fu-treated group exhibited pronounced immuno-expression of caspase-3 and TNF-α (Figs 3 and 4e and f). Fu/L.S.O. and L.S.O./Fu exhibited a notable reduction in the immuno-expression of both markers (Figs 3 and 4g–j).

## Discussion

Furan presents a risk to human health because to potential significant exposure via diet and airborne routes. There are limited data on Fu’s detrimental effects on the immune system. This study evaluated the immune-protective effects of *Lagenaria siceraria* supplementation against toxicity induced by Fu. This study found significant increases in ALT, AST, and LDH in rats exposed to furan. LSO supplementation leads to a significant restoration of the aged biomarkers, particularly in the protective agents. The therapy group’s ALT level did not significantly decline, although the protective groups did. Nonetheless, in both the therapeutic and protective cohorts, AST was significantly diminished to align with the control value. The hepatoprotective activity of *Lagenaria siceraria* extracts was assessed through the examination of liver function biochemical markers [34, 35]. Serum AST, ALT, and ALP activity markedly decreased in rats received lss, or silymarin as concurrent with CCL4. This study corroborates prior research by [36], which demonstrated that the oral administration of *Lagenaria siceraria* fruit (LSS) ethanolic extract to various groups of rats decreased blood ALT, AST, and ALP levels [37] For CCl4-induced liver damage, serum albumin levels; total protein, globulin, and the A/G ratio all exhibited a rise, indicating the hepatoprotective advantage of LSS [36]. Selmanoglu et al. [38] demonstrated that oral administration of Fu resulted in a considerable reduction in the levels of albumin, globulin, and serum protein. The abnormalities may be attributed to hepatocyte injury, inflammation, and/or necrosis induced by toxic metabolites of Fu. Our experiment showed non-significant reduction in serum protein which may be due to changes in dose or duration of experiment.

IgM, NO levels, and lysozyme activity were all significantly lower in the serum of FU-exposed rats than in control rats, according to this study. The LSO-only treated group has shown normal levels of the previously indicated markers, much like the control group. A notable alteration in IgM levels was observed solely in the prophylactic group, whereas no change was detected in the therapeutically co-administered group when compared to FU-exposed rats. LSO supplementation significantly altered NO levels in both co-treated groups, with a sharper effect observed in the therapeutic regimen, which showed a non-significant difference compared to the control group. Furthermore, the co-treatment of FU-exposed rats with LSO did not produce a substantial alteration in lysozyme activity, exhibiting a nonsignificant difference when compared to the FU-intoxicated group in both regimens. Alam et al. [39] discovered that rats administered Fu exhibited dramatically reduced serum levels of IgM and IgG, suggesting that Fu and its deleterious metabolites diminish humoral immunity. This alteration may be partially elucidated by B-lymphocyte depletion or activity suppression (i.e., IL-4 is essential for immunoglobulin production).

Previous studies indicate that furan exposure disrupts reproduction by hindering spermatogenesis, leading to death in Leydig cells and the germ cell lining. The harmful impacts of furan on humans and animals render it a significant global issue [40]. The current investigation demonstrated the impact of furan on testicular tissue, revealing that FU-exposed rats exhibited a significant reduction in sperm count and viability, whereas the protected group displayed increased sperm count and viability. The quantity of abnormal epididymal sperm in the FU-treated groups was significantly greater than that observed in the control group. Serum concentrations of LH and testosterone were markedly reduced in FU-exposed animals compared to control rats. In contrast to the FU-exposed group, the LSO dosage mitigated the FU-induced reduction in testosterone levels, particularly in the protected group of rats. Furthermore, in contrast to the group subjected to FU, LSO was noted to restore the LH level in both the protective and therapeutic protocols.

The MDA level in the testes was considerably elevated following exposure to FU. Despite the substantial recovery of elevated MDA levels in both the protective (LSO/FU) and therapeutic (FU/LSO) groups, there was a concomitant reduction in testicular TAC, GSH, SOD, and CAT activities. Nevertheless, the GSH level and reduced CAT and SOD activities were maintained at low levels in the testes of both cotreated groups, yet did not significantly differ from control values. Rehman et al. [41] reported that the outcomes of the *in vitro* investigation indicated that testicular tissue exhibited reduced levels of the antioxidants CAT, SOD, and POD. Furan administration elevated lipid peroxidation (LPO) and reactive oxygen species (ROS) levels in testicular tissues compared to the control group. These results align with prior studies indicating that exposure to furan and acrylamide elevated cellular levels of reactive oxygen species and lipid peroxidation. Elevated doses of furan (20 and 40 mg kg^-1^) reduce the diameter of the seminiferous tubule lumen, the height of the epithelium, and the sperm count within the epididymal lumen, hence augmenting reactive oxygen species (ROS) and oxidative damage in testicular tissues. An excessive generation of ROS may be responsible for all these changes. Molecules possessing unbound unpaired electrons that include oxygen and exhibit high reactivity. Furthermore, specific nonradical molecules are denoted as ROS. During normal metabolic processes, these compounds generate free oxygen ions. Reactive oxygen species (ROS) are generated during oxidative phosphorylation in the mitochondria [42, 43].

Our results showed similar histological damage as FU-Treated group exhibited testicular degeneration with necrosis of spermatogenic cells and coagulation of sperms within the seminiferous tubules.

The decrease in antioxidant enzyme levels diminishes the cell’s ability to neutralize the effects of reactive oxygen species by compromising its sensitivity to oxidative stress [44]. Furan has been identified as a genotoxic, cytotoxic, and apoptotic inducer. The degradation of proteins and lipids resulting from oxidative stress has been previously shown in prior studies [45]. We found that rats treated with FU had significantly lower levels of antioxidant enzymes, which maximizes the degenerative plateu of FU.

Rawi et al. [46] assert that lipids play essential structural and functional roles in numerous organs and cells inside the body. They are essential for the sustenance of physiological functions. Dyslipidemia, characterized by elevated levels of triglycerides, cholesterol, and fat phospholipids, refers to the disturbance in serum lipid concentrations and lipid profiles induced by oxidative stress. The LDH level in the current study exhibited a tendency towards normalcy and was considerably reduced in the two co-exposed groups compared to the FU-exposed group. No variations in protein content were observed. FU exposure significantly elevated LDL and total cholesterol values. The increase diminished with the administration of LSO, and it significantly differed from the Fu-exposed group in both protective and therapeutic protocols. Nonetheless, no substantial difference was observed in TG, HDL, or VLDL values across all treated groups. FU exposure markedly elevated LDL and total cholesterol levels in comparison to the control group. In both preventative and therapeutic regimens, this rise was markedly distinct from the Fu-exposed group and diminished with the administration of LSO. Rehman et al. [41] demonstrated that animal’s administered furan exhibited reduced HDL levels and elevated plasma total cholesterol, triglycerides, and LDL levels.

Furthermore, prior studies demonstrate that the administration of furan and acrylamide leads to a reduction in HDL and an elevation in plasma LDL, total cholesterol, and triglycerides [47, 48]. Previous research indicates that male infertility, low testosterone, and abnormal sperm morphology are correlated with increased cholesterol and reduced HDL levels [49]. Uzunhisarcikli et al. [50] previously reported analogous findings, indicating that a reduction in testosterone levels resulted in a reduced sperm count. Reproductive hormones govern spermatogenesis and intercellular interactions within the testis; hence, spermatozoa subjected to elevated levels of reactive oxygen species (ROS) exhibited diminished viability and motility, ultimately resulting in infertility [51].The current study’s histological findings indicated that the fu-treated group exhibited testicular degeneration, characterized by a diminished quantity of spermatogenic cells in the seminiferous tubules, with degeneration and necrosis of additional spermatogenic cells. This group had interstitial tissue edema between seminiferous tubules, congestion and edema in the tunica albuginea, as well as sperm coagulation and degeneration within the seminiferous tubules. The Fu/L.S.O.-treated group exhibited minor interstitial edema and congestion, moderate congestion of blood vessels in the tunica albuginea, and degeneration and necrosis of a limited number of seminiferous tubules.

Normal seminiferous tubules with little interstitial edema and congestion were noted in the LSO/Fu group. Rehman et al. [41] demonstrated a dose-dependent reduction in epithelial height and an increase in the diameter of seminiferous tubules and lumen. Administration of high dosages of furan resulted in seminiferous tubules exhibiting hollow lumens and reduced interstitial spaces. Previous studies indicate that reduced testosterone levels may correlate with a decrease in the height of the seminiferous epithelium, and diminished testosterone levels lead to a reduction in germ cells throughout stages VII to VIII of the spermatogenic cycle [52]. Rehman et al. [41] demonstrated that groups subjected to elevated doses of furan exhibited a decline in testosterone levels. Nonetheless, a decrease in testosterone levels is indicative of chemical toxicity [53]. As previously stated, oxidative stress results in diminished testosterone levels. This pertains to the principal role of antioxidant enzymes in Leydig cells and heightens the vulnerability of spermatogenesis to oxidative stress [54, 55]. The current investigation indicates that Furan induces oxidative stress, altering testosterone synthesis in the testes and diminishing sperm generation, finally resulting in spermatogenic arrest. On the other hand, treatment with LSO in both co-treated groups mitigated the oxidative stress plateau and increased testosterone levels, leading to improved seminiferous tubule health. These groups exhibited normal seminiferous tubules, with moderate interstitial edema and congestion (Fig 1). This improvement is also attributed to the enhancement of oxidative enzymes in these groups.

We report that exposure to furan markedly elevated the inflammatory biomarkers TNF-α and IL-6. The significance of mitogen-activated protein kinase (MAPK) signaling pathways in regulating cell growth, survival, and apoptosis is well acknowledged. Environmental stresses and other chemicals activate the Jun N-terminal kinase (JNK) and p38 MAPK pathways [56]. Activated p38 MAPK may induce apoptosis by phosphorylating or indirectly suppressing pro-survival Bcl-2 proteins in reaction to cellular stress [57, 58]. In contrast, mitogenic stimuli such as growth factors and cytokines activate ERK, which is crucial for cell survival and proliferation. The stimuli and cell type significantly affect the function of MAPK signaling [59]. Although p38 MAPK and ERK were identified as activated post-NFD therapy, their roles in naphtho[1,2-b] furan-4,5-dione (NFD)-induced apoptosis remained unclear. Consequently, we examined the role of MAPK in NFD-induced apoptosis with p38 MAPK and extracellular-signal-regulated kinase (ERK) inhibitors, respectively. The inhibitor experiment revealed that the NFD-induced suppression of hepatocellular carcinoma (HCC) tumor cell proliferation is primarily reversed by inhibiting p38 MAPK activity. HepG2 and Huh-7, two more HCC tumor cell lines, exhibited analogous results, indicating that p38 MAPK activation was pervasive and essential for NFD-induced HCC tumor cell death. NF-kB regulates genes associated with cell survival, proliferation, and apoptosis [60].

## Conclusions

This study shown that the antioxidant and liver-protective qualities of *Lagenaria siceraria* seed oil (LSO) prevent oxidative stress and organ damage in rats and alleviate furan-induced toxicity. The results propose an alternative to conventional treatments by positioning LSO as a possible natural therapy for furan toxicity. To elucidate its mechanisms and assess its efficacy in larger models, more research is required. All things considered, the study shows how beneficial plant-based remedies are for controlling exposure to environmental toxins and paves the way for potential future treatments.
